# An Interesting Case of Isolated False-Reactive Hepatitis B Surface Antigen

**DOI:** 10.1155/2021/9928098

**Published:** 2021-07-19

**Authors:** Victoria Costa, Zhen Zhao, Sabrina E. Racine-Brzostek, Gadi Lalazar, He S. Yang

**Affiliations:** ^1^Department of Pathology and Laboratory Medicine, New York Presbyterian Hospital, Weill Cornell Medicine, New York, NY, USA; ^2^Department of Gastroenterology and Hepatology, New York Presbyterian Hospital, Weill Cornell Medicine, New York, NY, USA

## Abstract

The standard serologic markers used to diagnose hepatitis B infection include hepatitis B surface antigen (HBsAg), hepatitis B surface antibody (anti-HBs), total hepatitis B core antibody (anti-HBc), and IgM antibody to hepatitis B core antigen (IgM anti-HBc). Different markers or combinations of markers are used to identify different phases of HBV infection and determine whether a patient has acute or chronic infection or immunity due to prior infection or vaccination or is seronegative and susceptible to future infection. Isolated HBsAg seropositivity is a peculiar serological pattern that requires investigation. Herein, we present a case of an asymptomatic female without a history of liver disease or evident risk factors for hepatitis, who underwent screening for infectious disease prior to resection of basal cell carcinoma involving her eyelid. The patient's laboratory testing showed positivity for HBsAg and the HIV 1/2 screen. To investigate, we performed serial dilutions, utilized heterophilicantibody blocking tubes, and repeated analysis using a different commercial assay (Abbott Architect i2000), all in support of a false-positive result attributed to a heterophilic antibody. Hence, we demonstrate that heterophilic antibody interference can result in isolated HBsAg positivity and recommend considering this form of interference in the differential where there is low clinical suspicion for viral infection.

## 1. Introduction

Hepatitis B virus (HBV) is transmitted through contact with bodily fluids, including through sexual contact, contaminated blood, or from an infected mother to her newborn. HBsAg is one of the first serum markers to appear during the course of acute HBV infection, and it is also a useful follow-up marker, since declining concentrations are observed in resolving hepatitis B virus infection. During the acute phase of infection, hepatitis B e-antigen (HBeAg) appears shortly after the appearance of HBsAg and, in some patients, disappears within several weeks as the acute infection resolves. Anti-HBc IgM antibodies are detectable at the outset of the clinical disease and gradually decline as the infection evolves. Anti-HBs antibodies become positive late in convalescence, between 6 weeks and 6 months, after HBsAg clearance. This antigen and antibody dynamic pattern occurs in the majority of HBV infections [1]. Isolated HBsAg seropositivity, where HBsAg is the only positive serologic marker within the hepatitis B panel, may be seen in the very early stage of HBV infection when HBeAg and anti-HBc IgM have not appeared yet and a patient has not shown any symptoms [1]. Transient HBsAg has also been observed in patients for up to 2 weeks after HBV vaccination [2, 3]. Other possibilities of isolated positive HBsAg may include host incompetence or HBV variants with probably mutation in the S-region [4]. In addition, it has been reported that a patient with lupus nephritis exhibited isolated HBsAg positivity [5]. Furthermore, potentially false-reactive HBsAg could be due to a range of autoimmune conditions, infection with a range of community-acquired viral or bacterial infections, and possible reactivation of latent infections such as herpes viruses or *H. pylori*.

Here, we report a case of a female patient with persistent isolated HBsAg positivity with a lack of symptoms, other serological markers, risk factors, or vaccination to explain the positivity, highly suggestive of a false-positive result requiring thorough investigation to evaluate potential interferences.

## 2. Case Report

A 77-year-old female with a past medical history significant for type 2 diabetes mellitus, hypertension, ischemic heart disease status after percutaneous intervention with stent placement, and cardiac ablation presented for evaluation of a positive HBsAg blood test. Several months prior to her presentation, the patient developed a basal cell carcinoma (BCC) of her right lower eyelid. During the patient's Mohs surgery, a common type of procedure performed in stages for carcinoma of the skin, to remove the BCC, a healthcare provider experienced a needle stick injury, which prompted infectious disease testing. The patient's postexposure laboratory testing revealed a positive hepatitis B surface antigen (HBsAg), a negative hepatitis B surface antibody (HBsAb), a negative hepatitis B core antibody (HBcAb), and an undetectable hepatitis B viral load (lower limit of detection 20 IU/mL), as well as a negative hepatitis A antibody and a negative hepatitis C antibody. The hepatitis panel was performed on the ADVIA Centaur XP analyzer (Siemens Healthineers, Erlangen, Germany). The HBsAg index value (IV) of this sample was 76.4 (cutoff IV = 1.0), considered to be reactive and above the threshold for confirmatory testing (IV = 50), so further testing was not performed. Hepatitis B e-antigen and antibody were sent out to a reference laboratory, and results were also negative. A rapid screening test for human immunodeficiency virus (HIV)1/2 antibodies and p24 antigen was positive (IV = 8.5, cutoff IV = 1) on the Siemens ADVIA Centaur XP, whereas the confirmatory assay (Geenius HIV 1-2 Supplemental Assay, Biorad, Hercules, CA, USA) for both HIV-1 and 2 was negative. The HIV-1 RNA qualitative test was sent out to a reference lab, and the result was negative. Liver function testing of this patient was within the normal range, including an alanine aminotransferase of <9 U/L, aspartate aminotransferase of 14 U/L, alkaline phosphatase of 80 U/L, and total bilirubin of 0.5 mg/dL.

Upon further review of the patient's history, no recognized, self-reported risk factors for viral hepatitis including unprotected sex, blood transfusions, tattoos, or intravenous drug abuse were reported. Furthermore, the patient denied personal or family history of liver disease or jaundice. Physical examination did not reveal stigmata of liver disease. Considering the patient's low risk for blood-borne infections, it was suspected that the HBsAg and HIV screening results might be false positives due to an unknown interference with the analytical assays.

The patient underwent repeat testing on the same instrument three weeks later. Both HBsAg and HIV screening tests remained positive, excluding accidental lab error or a transient contaminant as the cause of her positive results. Other hepatitis B antigens and antibodies remained negative. Both the HBsAg neutralization test and HIV 1-2 supplemental assay were negative.

Two- and four-fold dilutions of the new specimen were performed for the HBsAg. The HBsAg initial IV was 49.1 and then 19.7 with a two-fold dilution and 8.6 with a four-fold dilution. These nonlinear readings indicated the presence of an interfering substance in the specimen. Due to a suspicion of a heterophile antibody causing the interference, the samples were rerun in tubes containing a heterophilic antibody blocking reagent (Scantibodies Laboratory Inc., lot number LSFF0041B), resulting in negative HBsAg (IV < 0.10) and HIV (IV = 0.84) results. Our investigation led to the conclusion that heterophilic antibody interference may be the root cause of the false-positive HBsAg and HIV screening, especially since this antibody did not interfere with the HBsAg neutralization test and the HIV 1-2 confirmatory assay.

In order to better understand the interference of the heterophilic antibody with different testing platforms, the patient's serum specimens, with and without treatment with the heterophilic antibody blocking reagent, were sent to another laboratory which uses the Abbott Architect i2000 immunoassay analyzers (Abbott Park, Illinois, USA). Both HBsAg and HIV screening results were negative on Architect i2000, even in the absence of the heterophilic antibody blocking reagent.

The patient was retested 3 months after her Mohs surgery. The HBsAg and HIV screening tests remained positive. This study has been approved by the institutional review board (19-10020914) of Weill Cornell Medicine.

## 3. Discussion

Serologic testing of hepatitis B markers is vital to the diagnosis of infection and management. It is essential that these laboratory results are interpreted within the relevant clinical context. In this case, the lack of other positive serological markers and lack of risk factors or vaccination highly suggested a false-positive result early on in the workup of this patient.

This HBsAg assay is a two-site sandwich immunoassay using direct, chemiluminometric technology. The biotinylated anti-HBs mouse monoclonal antibody captures the HBsAg, forming HBsAg-antibody complexes, which are captured by the streptavidin-coated magnetic latex particles. A light signal is generated with the addition of a second acridinium-ester-labeled anti-HBs mouse monoclonal antibody. The amount of relative light units (RLUs) is directly proportional to the amount of HBsAg present in the patient sample. Heterophilic antibodies in the patient's serum are polyreactive antibodies that can bridge the two reagent antibodies and generate light signal without the presence of HBsAg. Heterophilic antibodies have been known to cause either false-positive (e.g., HIV [6]) or falsely elevated (e.g., prostate-specific antigen [7]) immunoassay results. The two-site sandwich immunoassays are particularly susceptible to this interference [8]. In light of this interference, most commercial assays over the last decade have begun incorporating blocking reagents against heterophilic antibodies in their assay reagent formulation to reduce heterophilic antibody interference, as demonstrated with the Abbott Architect immunoassay platform.

Isolated HBsAg seropositivity is a peculiar serological pattern. As such, screening results should be confirmed by an HBsAg neutralization test before the final results are reported. In this test, samples are incubated with anti-HBs antibody. If HBsAg is truly present, the anti-HBs antibody in the neutralization step blocks HBsAg, inhibiting its binding to the reagent antibodies and, consequently, reducing or eliminating its signal (positive neutralization test result) [1]. False-positive HIV 1/2/p24 screening results can also be identified by the HIV 1/2 supplemental assay which detects and differentiates individual antibodies to HIV-1 and HIV-2 in blood, as suggested by the current Centers for Disease Control and Prevention (CDC) recommendation for laboratory testing for the diagnosis of HIV infection [9].

False positivity for HBsAg has been reported in the presence of tumors, with a similar case of isolated HBsAg positivity identified in a patient with a parathyroid adenoma [2]. Our case is the first reported case in the literature of an isolated positive HBsAg in the setting of basal cell carcinoma. However, whether the presence of the heterophile antibody is due to the basal cell carcinoma, as was initially suspected, is unclear as the heterophilic antibody has persisted for several months based on repeatedly positive testing. The cause of this stable heterophilic antibody is unknown, but there are a variety of possible causes for heterophile antibodies, such as infection, transfusion, or systemic disease. Epstein–Barr Virus (EBV) infection, as an example, often causes heterophilic antibodies that persist at low levels for up to 1 year [10]. Our main limitation here is our inability to specifically identify the reason for this persistent heterophilic antibody in this isolated scenario. It may be worthwhile to evaluate for the disappearance of the antibody in this patient on repeating laboratory testing for a prolonged length of time if possible. Determining the accuracy of HBV testing is essential, particularly for certain patient groups such as dialysis patients or those who are screened for viral infection prior to administration of immune suppressive therapy, chemotherapy, or organ transplantation. For example, patients with rheumatoid arthritis are screened for HBV before beginning rituximab therapy, and HBV serology cross reactivity with rheumatoid factors has been reported. Both the laboratory and clinicians need to be aware of the possibility of false-positive screening as these results can have serious implications, such as causing potentially effective treatment strategies to be avoided, social stigma, or initiating additional testing for contacts and family members [11].

Overall, in these cases with isolated HBsAg positivity and low clinical suspicion for hepatitis infection, one should consider following a systematic trouble-shooting algorithm, including repeat testing, especially using a different commercial assay. This case demonstrates the importance of collaboration and open discussion between the clinician and the laboratory to recognize potential discrepancies of laboratory testing in the context of clinical correlation, all leading to better patient care.

## Data Availability

This case report involved a single patient whose data will remain restricted in accordance with the Health Insurance Portability and Accountability Act.
